# Mutation in the *COL2A1* gene is associated with acetabular dysplasia

**DOI:** 10.3389/fgene.2024.1521412

**Published:** 2025-01-20

**Authors:** Miaomiao Xin, Xin Guan, Jiangfei Yang, Yi Li, Zhentao Man, Hongsheng Sun, Min Fu

**Affiliations:** ^1^ Department of Rheumatology and Immunology, Shandong Provincial Hospital Affiliated to Shandong First Medical University (Shandong Provincial Hospital), Jinan, Shandong, China; ^2^ Department of Rheumatology and Immunology, Xing’an League People’s Hospital, Ulan Hot, China; ^3^ Department of Radiology, Shandong Provincial Hospital Affiliated to Shandong First Medical University (Shandong Provincial Hospital), Jinan, Shandong, China; ^4^ Department of Joint Surgery and Sports Medicine, Shandong Provincial Hospital Affiliated to Shandong First Medical University (Shandong Provincial Hospital), Jinan, Shandong, China

**Keywords:** *COL2A1*, mutation, synovial chondromatosis, developmental dysplasia of the hip, the hip joints

## Abstract

**Background:**

Developmental dysplasia of the hip (DDH) is one of the most common developmental disorders worldwide, caused by a combination of genetic and environmental factors.

**Methods:**

To investigate the genetic etiology of DDH in a proband (a 27-year-old male), we reviewed the patient’s clinical data and collected peripheral blood samples from the proband and his parents. Genomic DNA was extracted, and polymerase chain reaction (PCR) amplification was performed. Clinical whole-exome sequencing (WES) using next-generation sequencing (NGS) was conducted to identify potential mutation sites, which were then validated through Sanger sequencing. Bioinformatics analysis was performed to assess the pathogenicity of the identified variant, and 3D protein modeling was conducted to predict its impact on protein structure.

**Results:**

The proband presented with pain in bilateral hips, and based on clinical symptoms, laboratory findings and imaging studies, the final diagnosis was considered to be acetabular dysplasia with overlapping secondary synovial chondromatosis. Family history revealed similar symptoms in the proband’s father, while the grandparents and other family members were unaffected. The patient underwent bilateral total hip arthroplasty and synovectomy. NGS and Sanger sequencing identified a heterozygous missense mutation in the *COL2A1* gene (ex13, c.823C > T; p.Arg275Cys) in both the proband and his father, while this mutation was absent in the mother. Bioinformatic analysis indicated that the c.823C > T (p.Arg275Cys) variant is pathogenic, and structural modeling demonstrated that the substitution of arginine with cysteine at residue 275 altered the protein structure.

**Conclusion:**

Our findings highlight the diagnostic utility of NGS in identifying precise genetic causes of DDH. The identification of the *COL2A1* gene mutation in this present case represents a novel clinical phenotype, expanding the spectrum of disorders associated with *COL2A1* mutations.

## 1 Introduction

Developmental dysplasia of the hip (DDH) is a complex musculoskeletal disorder with a broad spectrum of clinical manifestations, ranging from asymptomatic cases with mild radiological abnormalities to more severe forms, including minor joint instability, acetabular dysplasia, subluxation, and complete dislocation of the hip joint (Basit et al., 2016). While mild dysplasia may resolve spontaneously, more severe hip deformities, if misdiagnosed or improperly treated during early stages, tend to worsen progressively, resulting in chronic hip pain, restricted movement, abnormal gait, and eventually degenerative arthritis in adulthood (Zhao et al., 2023).

DDH arises from an interplay of multiple genetic and environmental factors (Zhao et al., 2023). The inheritance pattern of DDH is generally thought to follow an autosomal incomplete dominant model (Zhao et al., 2023). However, the significant genetic heterogeneity observed among DDH cases means that the condition does not adhere to classical Mendelian inheritance (Basit et al., 2017). Current genetic studies on DDH have focused primarily on specific chromosomal regions, particularly chromosomes 1, 3, 12, 17, and 20 (Kenanidis et al., 2020). Several genes have been implicated in the pathogenesis of DDH, including C-X3-C motif chemokine receptor 1 (*CX3CR1*), growth differentiation factor 5 (*GDF5*), collagen type I alpha 1 chain (*COL1A1*), Asporin (*ASPN*), and vitamin D receptor (*VDR*) (Zamborsky et al., 2019). Additionally, interleukin 6, homeobox genes (*HOXD9* and *HOXB9*), pregnancy-associated plasma protein A2 (*PAPPA2*), transforming growth factor-beta 1 (*TGF-β1*), T-box transcription factor (*TBX4*), ubiquinol-cytochrome C reductase complex chaperone (*UQCC*), ribosome biogenesis factor (*BMS1*), and fibroblast growth factor 2 (*FGF2*) have also been linked to the development of DDH (Zamborsky et al., 2019).

Environmental risk factors associated with DDH include breech position, female gender, family history of DDH, first-born, physical limitations within the uterus (macrosomia, oligohydramnios, multiple pregnancies), and incorrect swaddling position (swaddling infants in the adducted and extended position) (Nandhagopal et al., 2024; Ortiz-Neira et al., 2012).

The *COL2A1* gene, located on chromosome 12q13.11, contains 54 exons and encodes the α1 chain of type II procollagen (Barat-Houari et al., 2016). Three pro-α1 chains intertwine to form the procollagen molecule, which undergoes further processing into fibrils that are then cross-linked to form mature type II collagen (Col-II) fibers, which are important components of cartilage structure and function (Barat-Houari et al., 2016).

Mutations in the *COL2A1* gene give rise to a diverse group of disorders collectively termed type II collagenopathies. Zhang et al. summarized the relationship between *COL2A1* gene mutations and type II collagenopathy phenotypes, finding that N-propeptide regional mutations (especially exon 2) lead to mild phenotypes, while C-propeptide regional mutations cause severe and lethal phenotype (Zhang et al., 2020). Non-substitutions mutations (such as deletions, duplications, and insertions) are more likely to lead to mild phenotypes, except for small deletion mutations, which, unless causing frameshifts, typically result in severe or lethal phenotypes (Zhang et al., 2020).

Research on the relationship between *COL2A1* and acetabular dysplasia, including case reports and genetic linkage analyses related to hip joint involvement in type II collagenopathies, has shown that *COL2A1* is a key player. Namaqualand Hip Dysplasia (NHD) represents a milder form of spondyloepiphyseal dysplasia (SED), characterized primarily by progressive arthropathy of the hip joint (Agenbag et al., 2020). A linkage analysis spanning four generations within a South American family indicated an association between NHD and *COL2A1* gene (Sher et al., 1991). Further supporting this connection, Agenda et al. discovered a missense pathogenic variant, c.2014G > T, in the *COL2A1* gene among 23 individuals from a five-generation South African family affected by NHD (Agenbag et al., 2020). This finding underscores that mutations in the *COL2A1* gene are directly linked to the development of the NHD phenotype. Additionally, Granch and colleagues have demonstrated that polymorphisms in the *COL2A1* gene are also associated with osteoarthritis (OA) secondary to DDH (Granchi et al., 2002).

In the study of the pathogenesis of DDH, COL2A1 is also used as an indicator to evaluate cartilage metabolic capacity. Feng et al. investigated the relationship between DDH progression and cartilage metabolism disorders by analyzing cartilage tissues from patients with DDH, OA, and femoral neck fractures using histochemical staining, immunohistochemistry, and Western blot methods. Their findings revealed that the levels of aggrecan and Col-II in the DDH group were significantly lower than those in the control group and even lower than those in the OA group. Additionally, collagenous fibrils in the DDH group appeared highly rare and obscure (Feng et al., 2017). Normal articular cartilage is characterized by abundant aggrecan and Col-II, which may provide osmotic function and mechanical support, enabling the cartilage to resist compression (Feng et al., 2017). The reduction and disorganization of aggrecan and Col-II in DDH patients can lead to further joint cartilage degeneration and structural lesions, which would probably result in an articular cartilage metabolic disorder and hip joint dysfunction (Feng et al., 2017).

Here, we report a case of acetabular dysplasia accompanied by secondary synovial chondromatosis (SSC), whose father also suffers from the same conditions. To investigate the underlying etiology of these conditions, whole-exome sequencing (WES) was performed in the proband, revealing a *COL2A1* mutation. This report also reviews recent progress in the genetic understanding of these disorders.

## 2 Materials and methods

### 2.1 Compliance with ethical standards

This study was approved by the Medical Ethics Committee of Shandong Provincial Hospital (SWYX:2022-449). Clinical and laboratory procedures were conducted after obtaining written informed consent from all participants. All procedures adhered to the principles outlined in the Helsinki Declaration.

### 2.2 Next-generation sequencing (NGS) and variant calling

Peripheral blood DNA extraction: Peripheral blood samples were collected from the proband and his parents using EDTA-anticoagulated tubes. Genomic DNA was extracted from the samples using the QIAamp DNA Blood Midi KIT (Qiagen, Germany) in accordance with the manufacturer’s instructions and quantified using both a NanoDrop spectrophotometer and a Qubit fluorometer (Thermo Fisher Scientific, Waltham, MA, United States).

NGS analysis: WES was performed on the proband’s genomic DNA, which was sent to Hangzhou Dean Medical Laboratory Center for bioinformatics analysis. The analysis targeted coding regions of 5,075 genes associated with hereditary diseases, with a specific focus on genes linked to inherited bone diseases and chondrosarcoma. High-quality variants were identified through filtering and screening processes, followed by annotation using major genetic databases, including NCBI, dbSNP, OMIM, ESP, ExAC, ClinVar, and Thousand Genomes. The pathogenicity of identified variants was further evaluated using predictive bioinformatics tools such as SIFT, PolyPhen-2, and MutationTaster.

### 2.3 Sanger sequencing to verify the variant

Polymerase chain reaction (PCR) amplification and Sanger sequencing were conducted to confirm the candidate mutation. Primers were designed to target the identified mutation site, and PCR amplification was performed on the genomic region containing the suspected mutation. The PCR products were stored appropriately before being subjected to Sanger sequencing using an ABI 3130xl genetic analyzer. Sequencing results were aligned and compared with NCBI reference sequences using Chromas software to confirm the mutation.

### 2.4 Bioinformatic analysis

Conservation across species was assessed using the T-Coffee tool (https://www.ebi.ac.uk/jdispatcher/msa/tcoffee/). For the protein modeling, wild-type protein models were generated using the SWISS-MODEL tool (https://swissmodel.expasy.org/). The spatial structure and altered residue of the mutant protein were visualized using PyMOL software to assess the structural impact of the identified mutation.

## 3 Results

### 3.1 Clinical characteristics, diagnosis and treatment process

#### 3.1.1 The proband

The proband is a 27-year-old male who presented with bilateral hip pain lasting 6 months, which had progressively worsened over the past 2 months. Initially, the pain began following physical exertion and worsened with activity but did not resolve with rest. The patient reported no significant worsening at night. Associated symptoms included mild low back pain, but there was no heel pain, joint swelling, or deformity of small joints in the limbs. The patient sought treatment at a local hospital, where magnetic resonance imaging (MRI) of the lumbar spine revealed Schmorl’s nodes from T11 to L5 vertebrae. Based on these findings, the patient was diagnosed with seronegative spondyloarthropathy and prescribed oral non-steroidal anti-inflammatory drugs (NSAIDs), which provided partial symptom relief. However, 2 months prior to admission to our hospital, the bilateral hip pain worsened and he was admitted to our hospital.

Since the onset of the disease, the patient has reported no history of fever, cough, muscle soreness, headache, urinary urgency, frequent urination, abdominal pain, or ophthalmia. His body weight remained stable, and he denied smoking or alcohol consumption. Additionally, the patient had no history of chronic diseases, such as hypertension, diabetes, coronary heart disease, or cerebrovascular disease. He also denied any history of infectious diseases (e.g., hepatitis or tuberculosis), exposure to infectious individuals, psoriasis, or chronic diarrhea. There was no personal or family history of other significant medical conditions. The patient had received all routine childhood vaccinations. His parents were not consanguineous, but his father had experienced similar symptoms.

On physical examination, the patient’s vital signs were within normal ranges: body temperature, 36.9°C; heart rate, 87 beats per minute; respiratory rate, 21 breaths per minute; blood pressure, 140/82 mmHg; and oxygen saturation, 99% on ambient air. The patient appeared well-developed and well-nourished, although his gait was impaired. No abnormalities were observed in his conjunctiva or vision, and mucosal surfaces were dry without evidence of oral, vulvar, or perianal ulcers. Skin examination revealed no rashes or lymphadenopathy. Cardiopulmonary and abdominal examinations were unremarkable. Upon musculoskeletal examination, the skin overlying the hips showed no redness, swelling, or ulceration, and the local temperature was normal. Moderate tenderness was detected in the anterior space of the left hip joint. Patrick’s test was positive bilaterally, suggesting hip joint pathology. Joint mobility was restricted on both sides, with ranges of motion measured at 100°flexion, 0° extension, 20° pronation, and 20° supination. Sensory function, muscle strength, and peripheral circulation were intact in both lower limbs, with palpable and adequate pulses in the bilateral dorsalis pedis arteries.

**FIGURE 1:**
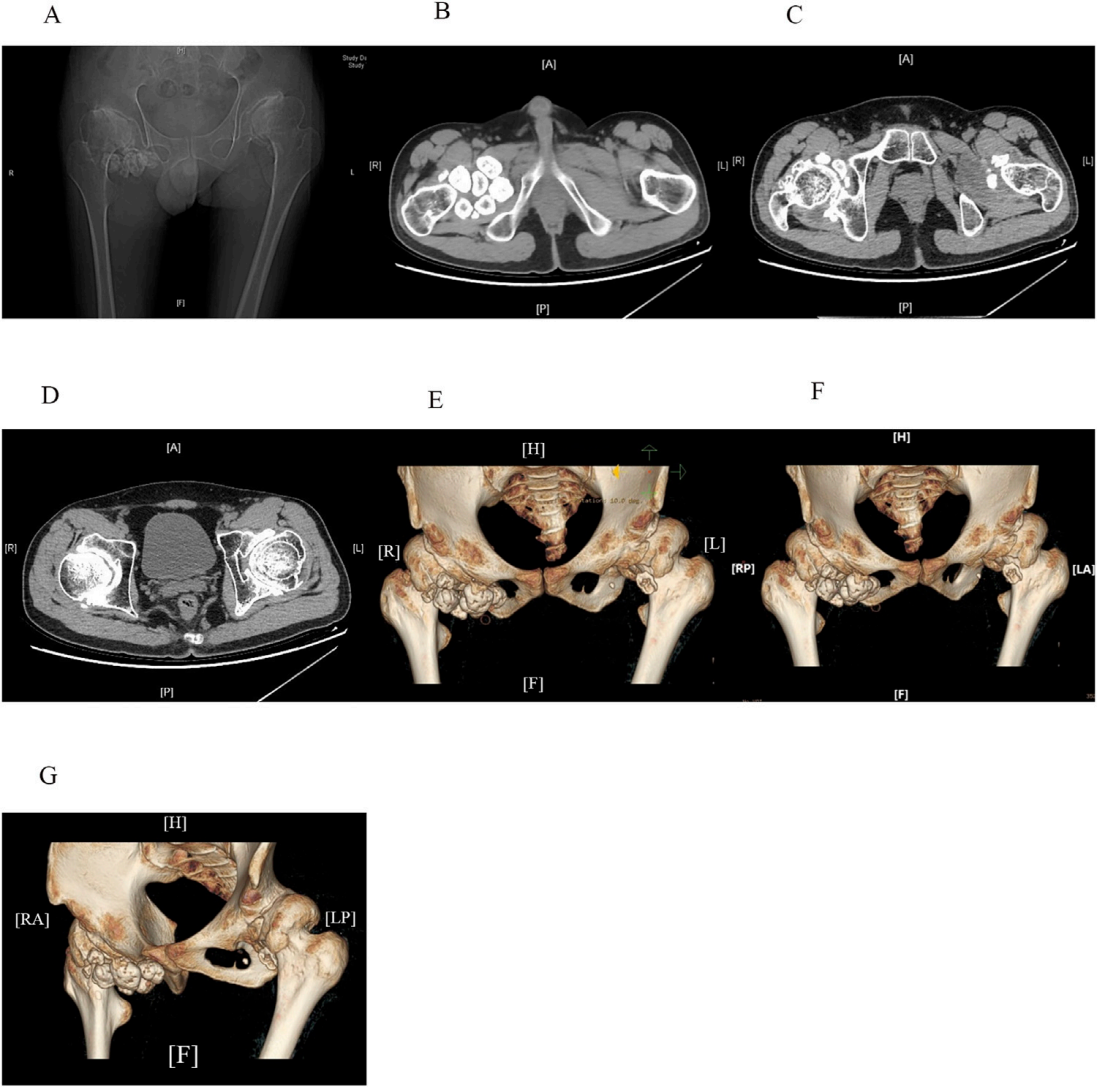
CT scans **(A–D)** and three-dimensional reconstruction images **(E–G)** of the hip joints reveal shallow acetabula with increased acetabular indices and bone hyperplasia along the joint margins bilaterally. The femoral heads appear hypertrophic and flattened, with anterolateral displacement and thick, short femoral necks. The neck-stem angle of the femur is increased, and the hip joint spaces are nonuniformly narrowed. Articular surface sclerosis and subarticular cystic changes are present on both sides. Enlargement of the bilateral hip joint capsules is evident, with multiple pomegranate seed-like high-density foci, particularly on the right side. Soft tissue swelling can also be observed around the joint.

**FIGURE 2:**
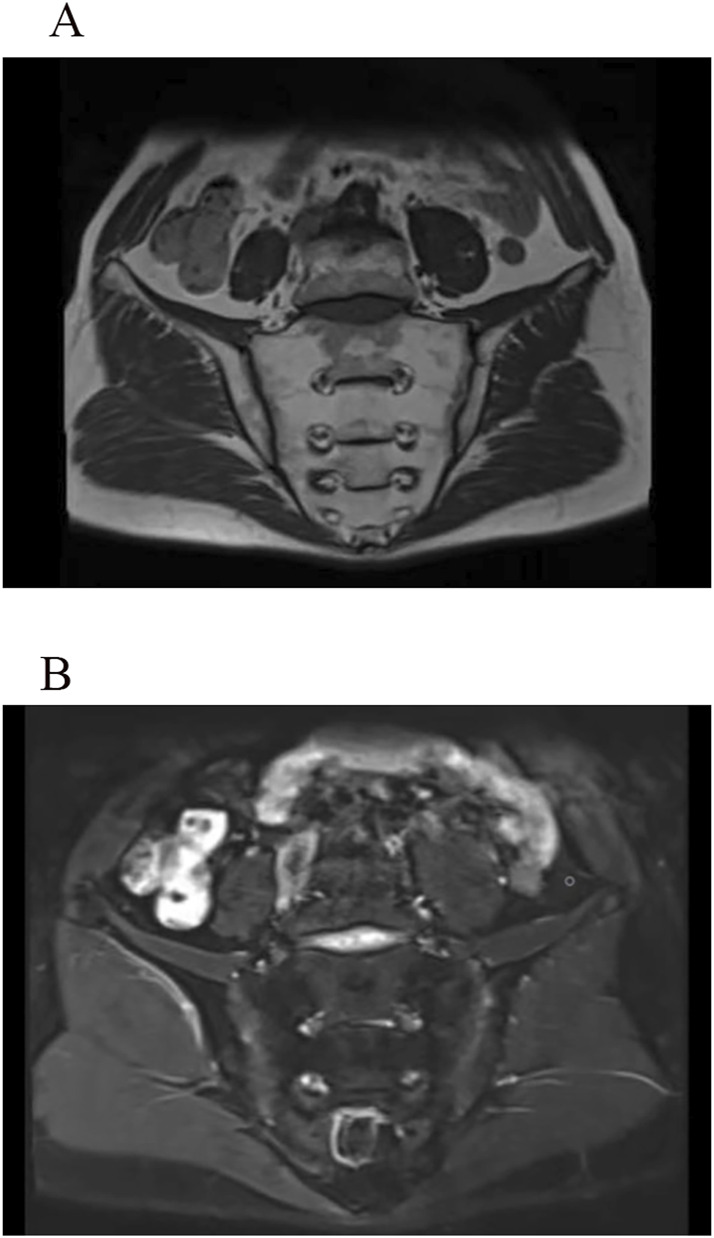
MRI images of the sacroiliac joints show long T1 **(A)** and T2 **(B)** signals on both the sacral and iliac surfaces. The articular surfaces appear smooth on T1WI, without signs of bone erosion, new bone formation, or abnormal fat deposition. The joint spaces are normal, with no evidence of pathological narrowing.

Comprehensive laboratory and imaging examinations were performed. Liver function, lactate dehydrogenase, creatine kinase, and biochemical test results were normal, except for alkaline phosphatase, which was elevated at 139 U/L (reference range: 45–125 U/L). Routine blood, urine and stool tests were unremarkable. Erythrocyte sedimentation rate, C-reactive protein, anti-streptolysin, and *Mycobacterium tuberculosis* T-cell detection tests showed no abnormalities. Complement 3, complement 4, and immunoglobulin levels were within normal limits. Bone metabolism evaluation revealed elevated total procollagen type I amino-terminal peptide at 128.40 ng/mL (reference range: 9.06–76.24 ng/mL) and a 25-dihydroxyvitamin D level of 6.62 ng/mL (reference range: 20–100 ng/mL). Testing for human leukocyte antigen (HLA)-B27 was negative. Autoimmune markers, including rheumatoid factor, anti-cyclic citrullinated peptide antibody, anti-RA33 antibody, antinuclear antibody, and anti-SA antibody, were all negative. Radiographic imaging identified several abnormalities. A plain X-ray of the pelvis showed dysplastic osteoarthrosis of the acetabulum. Full-length anteroposterior radiographs of both lower limbs demonstrated OA and SSC in the knees, with no abnormalities observed in the ankles. Computed tomography (CT) with three-dimensional reconstruction of the hip joints (Figure 1) confirmed bilateral dysplastic osteoarthrosis of the acetabulum and SC. MRI of the cervical spine indicated degenerative changes and intervertebral disc herniation at the C3/4 and C5/6 levels. MRI of the sacroiliac joints (Figure 2) initially suggested “bilateral sacroiliac arthritis.” However, following a review by two additional radiologists, the sacroiliac joint surfaces were considered smooth, and the edema signal did not support the diagnosis of sacroiliac arthritis. Based on the clinical presentation and imaging findings, the diagnosis of acetabular dysplasia with overlapping SSC was established.

**FIGURE 3:**
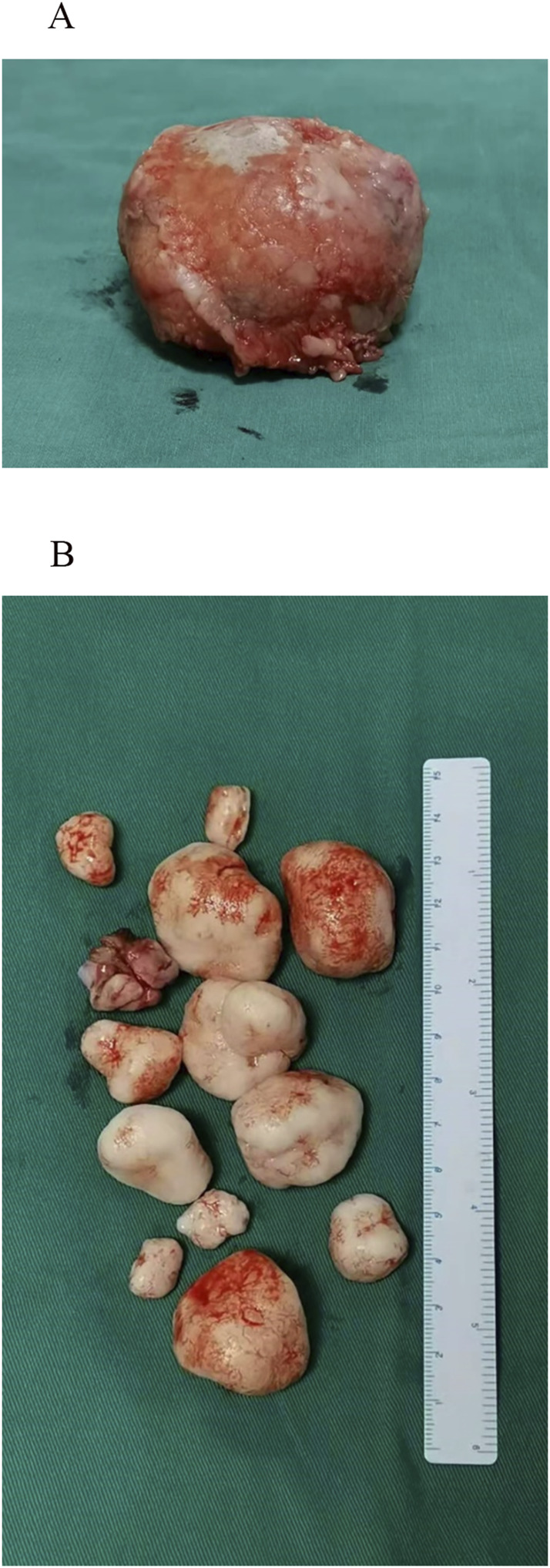
Intraoperative visualization. **(A)** A large, round-like tumor body observed during the procedure. **(B)** Multiple smooth, white or milky-white loose bodies of varying sizes, ranging from several millimeters to several centimeters in diameter, were identified during surgery.

After evaluation by osteoarticular surgeons, the patient was diagnosed with acetabular dysplasia accompanied by SSC. The patient’s general condition was assessed to be good, and no significant contraindications to surgery were identified based on the completed examinations. To alleviate pain, restore hip function and improve quality of life, bilateral total hip arthroplasty and synovectomy were performed under general anesthesia with endotracheal intubation. During the surgery, multiple smooth, loose bodies of varying sizes, with white or milky-white appearance, were identified, along with a large, rounded mass (Figure 3). Postoperatively, the patient received symptomatic and supportive care. Instructions were provided to maintain clean dressings, follow a nutrient-rich diet to support recovery, and perform exercises to enhance hip flexion. The patient was advised to practice dorsiflexion to prevent thrombosis and scheduled for follow-up in the Outpatient Department of Bone and Joint Surgery 1 month post-operation.

#### 3.1.2 The Proband’s father

**FIGURE 4:**
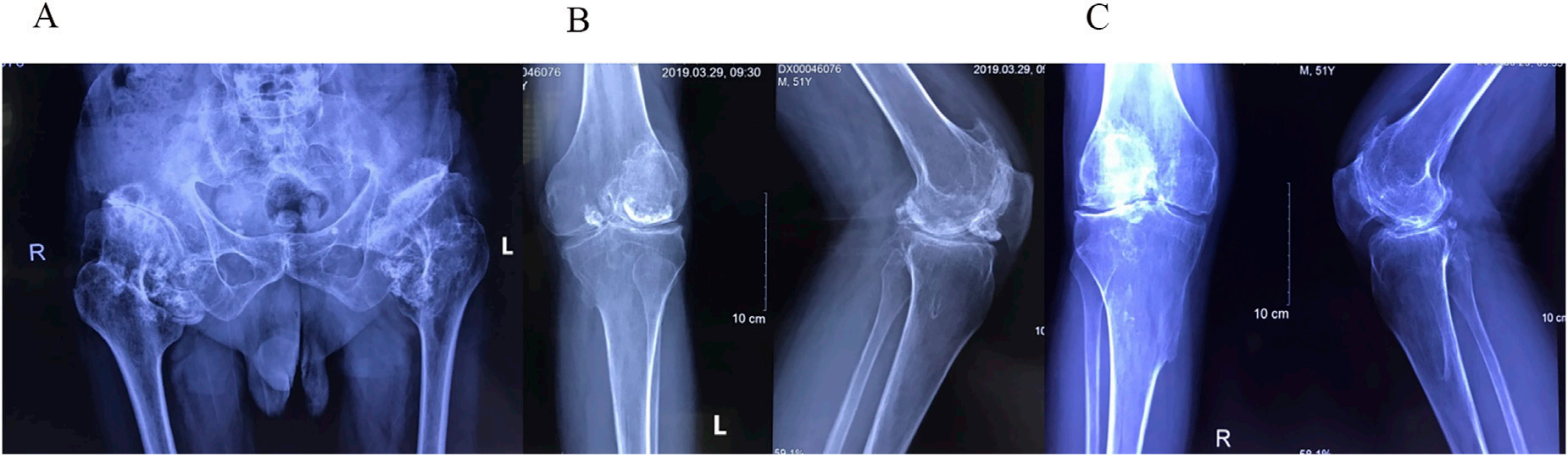
**(A)** Pelvic radiograph showing shallow acetabula with increased acetabular indices and bone hyperplasia along the joint margins bilaterally. The femoral heads appear hypertrophic and flattened with anterolateral displacement. The left hip joint demonstrates dislocation, indicated by overlapping of the femoral head and acetabular shadow, along with superior displacement of the femur. Both femoral necks are thick and short, and the neck-stem angles are increased bilaterally. The right hip joint space is unevenly narrowed, with sclerosis of the articular surface and degeneration of the subarticular capsule in the weight-bearing area. Enlargement of the bilateral hip joint capsules is observed, with multiple pomegranate seed-like high-density foci, particularly on the right side. Soft tissue swelling is present around the joint. **(B)** Anteroposterior radiograph of the left knee joint and **(C)** anteroposterior radiograph of the right knee joint show narrowing of the joint spaces, low patellar position (patella baja), and slight outward displacement of the patella on both sides. Bone hyperplasia and sclerosis of the joint surfaces are visible along the joint margins. Multiple pomegranate seed-like high-density foci of varying sizes are evident within the joint spaces, especially in the left knee joint, where a larger focus is located anteriorly. Bony protrusions are observed on the medial sides of the upper tibiae bilaterally. These protrusions are continuous with the tibial cortex and connected to the medullary cavity, consistent with osteochondromas.

The proband’s father also experienced similar symptoms of bilateral hip pain, but without restricted hip movement or a limp, indicating milder symptoms. He had never received a formal diagnosis or treatment. After the diagnosis of his son, the father underwent X-ray imaging of the pelvis and bilateral knees (Figure 4). The pelvic X-ray revealed dysplastic osteoarthrosis of the acetabulum, bilateral SSC, and dislocation of the left hip joint. Anteroposterior and lateral radiographs of both knees demonstrated OA, bilateral SC, and the presence of osteochondroma.

### 3.2 Genetic analysis

Through WES technology, we can identify all variants (including point mutations and small indels) in the exons of 5,075 genes and their adjacent ±20 bp intronic regions in the patient. The detection is performed using a standardized target region capture sequencing platform, with quality control metrics as follows: an average coverage of 98.99% and an average sequencing depth of 124.86. Subsequently, the identified variants are annotated and compared with disease-related databases such as Online Mendelian Inheritance in Man (OMIM)and Human Phenotype Ontology (HPO) to determine if they match known clinical phenotypes. This process helps us understand whether the discovered genetic variants are associated with specific diseases or symptoms.

**FIGURE 5:**
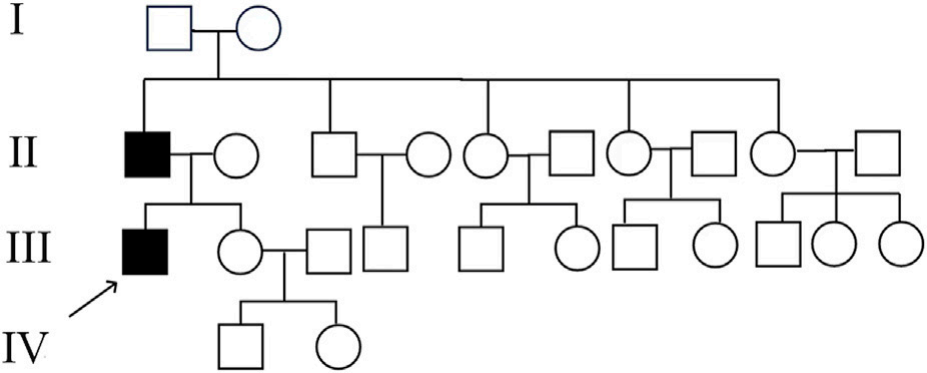
Pedigree of the proband’s family. Both the proband and his father were affected, while his grandparents and other family members did not exhibit similar symptoms.

**FIGURE 6:**
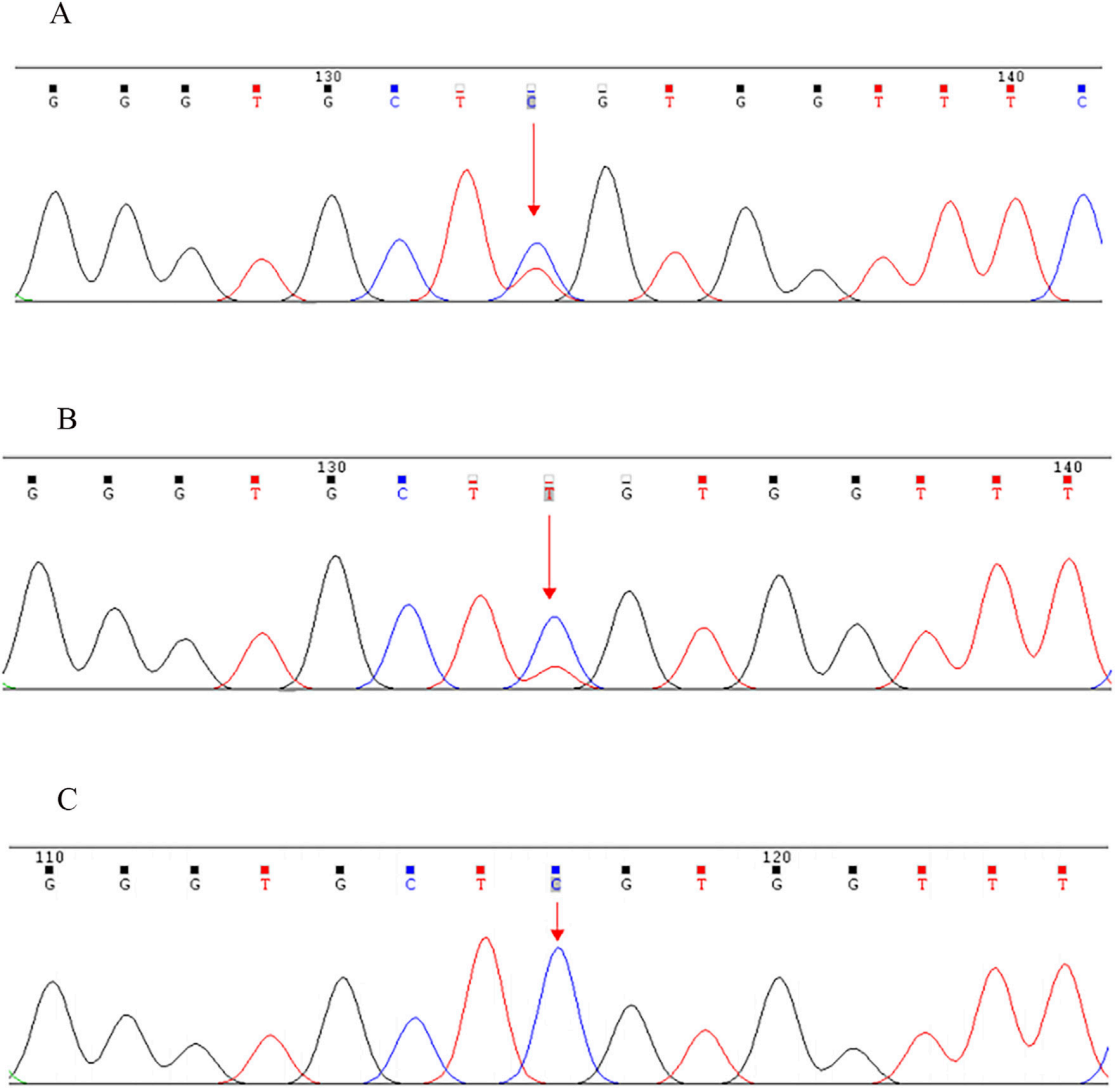
Sanger sequencing validation of the *COL2A1* exon 13 c.823C > T (p.Arg275Cys) mutation in the proband and his parents. **(A)** Proband (mutation detected), **(B)** Father (mutation detected), **(C)** Mother (mutation not detected). Note: NCBI reference sequence: GCA​GGG​TGC​TCG​TGG​TTT​CCC.

NGS revealed that the proband carried a pathogenic heterozygous missense mutation in the *COL2A1* gene, specifically in exon 13 at c.823C > T (p.Arg275Cys) (Table 1). No other pathogenic mutations, suspected pathogenic variants, or variants of unknown clinical significance were identified in genes related to chondrosarcoma. Sanger sequencing confirmed the presence of the same *COL2A1* mutation in both the proband and his father. The heterozygous missense mutation (c.823C > T) in exon 13 of the *COL2A1* gene resulted in the substitution of arginine with cysteine at the p.Arg275 position (p.Arg275Cys). The proband’s mother tested negative for this mutation (Table 2; Figures 5, 6).

**TABLE 1:** The results of clinical whole-exome high-throughput sequencing of the proband.

Gene	Nucleotide variation	Amino acid variation	Mutation type	Zygosity	Inheritance pattern	1,000 Genomes frequency/db138	Clinical significance
*COL2A1*_exon13NM_001844.4	c. 823C > T	p. Arg275Cys	missense mutation	heterozygous	AD	/rs121912876	pathogenic

Note: c. 823C > T indicates the replacement of cytosine with thymine at the 823rd nucleotide of the *COL2A1* gene. p. Arg275Cys represents the substitution of arginine with cysteine at the 275th amino acid position. AD, refers to autosomal dominant inheritance. The clinical significance is indicated as pathogenic, meaning the mutation is disease-causing.

**TABLE 2:** Fixed-site Sanger sequencing results for the proband and his parents for the *COL2A1* gene.

Scope of test	Test results		
Site	The first patient	His father	His mother
*COL2A1*_exon13 c. 823C > T (p. Arg275Cys)	Het	Het	N

Het means heterozygous mutation, N means no such mutation.

### 3.3 Bioinformatic analysis

**FIGURE 7:**
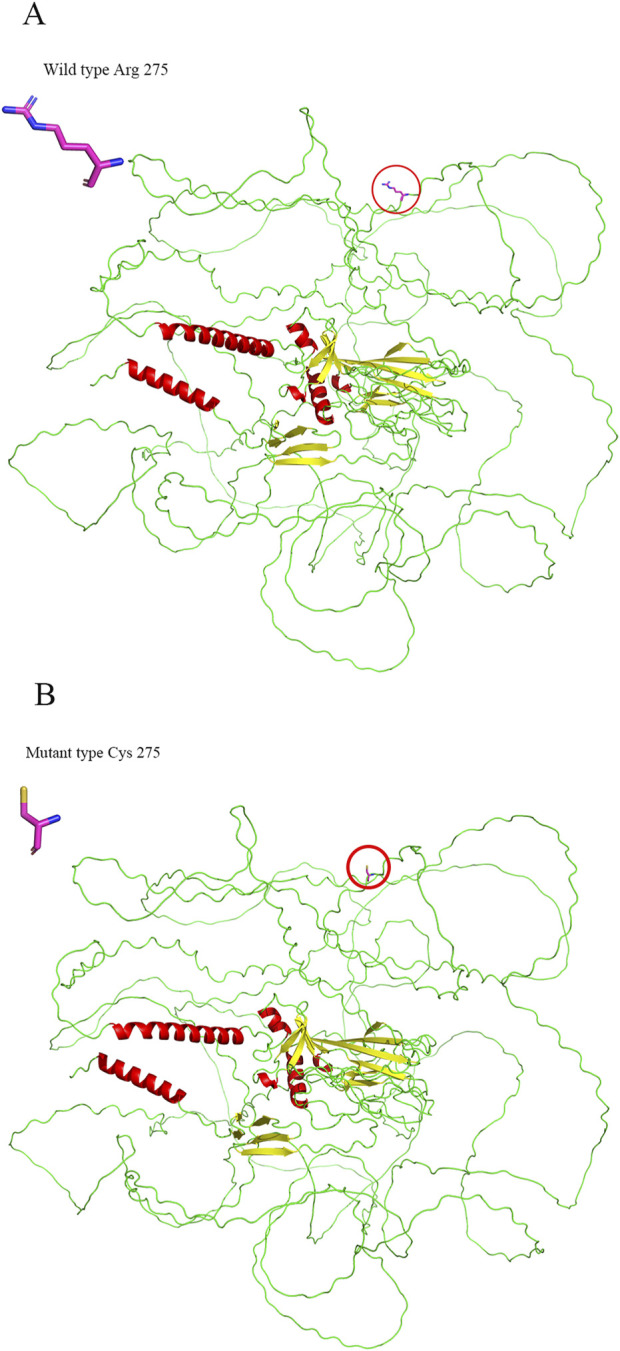
3D structural comparison of the wild-type **(A)** and mutant **(B)** p.Arg275Cys forms of COL2A1.

**FIGURE 8:**
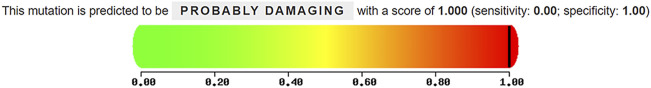
Pathogenicity prediction of the c.823C > T (p. Arg275Cys) missense mutation in *COL2A1* using PolyPhen-2.

Conservation analysis indicated that the arginine residue at position p.Arg275 is highly conserved across multiple species, underscoring its potential functional importance (Table 3). Structural modeling revealed that the substitution of arginine with cysteine at residue 275 led to alterations in the protein structure (Figure 7). The c.823C > T variant’s pathogenicity was assessed via bioinformatic tools, including SIFT, PolyPhen-2, and MutationTaster. Referring to the HumanDiv database, Polyphen-2 scores ≥0.957 suggest the amino acid substitution is probably damaging; scores between 0.453 and 0.956 indicate the substitution is possibly damaging; and scores ≤0.452 imply the substitution is benign. SIFT scores span from 0 to 1, with amino acid substitution scores < 0.05 indicating a prediction of deleteriousness, and scores ≥0.05 suggesting the substitution is tolerated. The c.823C > T (p. Arg275Cys) in the *COL2A1* gene was predicted to be deleterious: MutationTaster (0.999, disease-causing), SIFT (0.010, damaging), and PolyPhen-2 (1.000, probably damaging) (Figure 8).

**TABLE 3:** Conservation analysis of the arginine residue at site p.Arg275 in COL2A1.

Species	COL2A1 amino acid sequence									
	271	272	273	274	275	276	277	278	279	280
*Homo sapiens*	P	Q	G	A	R	G	F	P	G	T
*Bos taurus*	P	Q	G	A	R	G	F	P	G	T
*Canis lupus* familiaris	P	Q	G	A	R	G	F	P	G	T
*Rattus norvegicus*	P	Q	G	A	R	G	F	P	G	T
*Mus musculus*	P	Q	G	A	R	G	F	P	G	T
*Pan troglodytes*	P	Q	G	A	R	G	F	P	G	T
*Sus scrofa*	P	Q	G	A	R	G	F	P	G	T
*Ornithorhynchus anatinus*	P	Q	G	A	R	G	F	P	G	T
*Felis catus*	P	Q	G	A	R	G	F	P	G	T
*Gorilla gorilla gorilla*	P	Q	G	A	R	G	F	P	G	T

## 4 Discussion

Dysplastic osteoarthrosis of the acetabulum is a hip joint disorder that develops in adults due to long-term biomechanical abnormalities resulting from congenital acetabular dysplasia (Tian et al., 2003). Key pathological changes associated with this condition include secondary OA, subchondral pseudocysts, and chronic joint dislocations (Tian et al., 2003). Acetabular dysplasia is diagnosed based on specific radiographic criteria, including a shortened acetabular roof, a center-edge angle of less than 30°, with or without increased acetabular inclination (acetabular index > 45°), and femoral head exposure exceeding 25% on anteroposterior pelvic radiographs (Tian et al., 2003). Radiographic features include the presence of OA alongside a shallow or steep acetabulum (Tian et al., 2003). Early radiographic signs include thickening of the white line of the acetabular cap, resembling an “eyebrow,” with pointed or tilted outer margins (Tian et al., 2003). As the condition progresses, sclerosis of the acetabular roof enlarges, subchondral cystic changes develop, and joint space narrows within the weight-bearing region (Tian et al., 2003).

SC is characterized by the formation of multiple cartilaginous or osteochondral loose bodies within the synovium (Adelani et al., 2008). On X-ray and CT imaging, SC is typically identified by multiple high-density calcified foci within the joint (Alexander et al., 1987).

The radiographic findings in our patients were consistent with acetabular dysplasia and SC. The X-ray images revealed classic features of acetabular dysplasia, such as shallow and flattened acetabula, excessive tilt, and incomplete femoral head coverage. CT imaging demonstrated multiple pomegranate seed-like high-density foci in both hip joints, consistent with calcified cartilage nodules. Overall, these imaging findings provided sufficient evidence to establish a definitive diagnosis of acetabular dysplasia with overlapping SC.

Given the similarity of symptoms between the proband and his father, the possibility of a genetic etiology was investigated. Genetic testing identified a *COL2A1* gene mutation in both the proband and his father. Specifically, a heterozygous missense mutation (c.823C > T) was detected in exon 13 of the *COL2A1* gene, resulting in an amino acid substitution from arginine to cysteine at position 275 (p. Arg275Cys). The underlying pathogenesis may involve the mutation leading to congenital acetabular dysplasia, which subsequently initiates a cascade of pathological changes affecting both bone and soft tissues. These abnormalities alter the morphology and biomechanics of the hip joint, contributing to the development of SC and secondary arthritis, which in turn exacerbate joint damage and structural deterioration.

The clinical phenotype associated with the c.823C > T (p.Arg275Cys) mutation identified in this study differs from previously reported cases in the literature (Hildebrand, 2015; Matsui et al., 2009). The presence of acetabular dysplasia in these patients expands the phenotypic spectrum of *COL2A1* mutations. The p.Arg275Cys mutation in the *COL2A1* gene has been previously reported to manifest as spondyloepiphyseal dysplasia congenita (SEDC), spondyloarthritis, early-onset osteoarthritis (EO-OA), and Czech dysplasia (Hoornaert et al., 2006). The current focus is to distinguish these cases from other type II collagenopathies that present with overlapping clinical features. The following section discusses type II collagenopathies with clinical presentations and mutation sites similar to those observed in these patients.

### 4.1 Discussion on type II collagenopathies

#### 4.1.1 SED

SED refers to a group of rare genetic skeletal disorders primarily affecting the spine and epiphyses, with the predominant clinical features including disproportionate short stature, thoracocyllosis, and progressive degeneration of multiple joints (Liu, 2014). Radiographic findings often reveal platyspondyly, epiphyseal dysplasia, and narrowed joint spaces (Liu, 2014). Inheritance patterns of SED include autosomal dominant, autosomal recessive, and X-linked recessive modes (Liu, 2014). According to the Nosology and Classification of Genetic Skeletal Disorders (2010 revision), SED is categorized into nine clinical types, which include SEDC and Czech dysplasia, among others (Warman et al., 2011).

##### 4.1.1.1 SEDC

SEDC predominantly affects the vertebrae and the proximal epiphyses of long bones (Liu, 2014). Clinical manifestations in patients with SEDC are diverse and include short stature and skeletal deformities (Li et al., 2015). Common spinal abnormalities include scoliosis and platyspondyly (flattened vertebral bodies) (Li et al., 2015). Additionally, atlantoaxial instability, often resulting from odontoid hypoplasia, may lead to cervical cord compression (Al Kaissi et al., 2019; Tofield and Mackinnon, 2003). Hip deformities, such as avascular necrosis-like changes in the acetabulum and bilateral femoral epiphyses, are frequent and may cause hip pain and reduced walking endurance (Li et al., 2015). Patients with SEDC may also present with hearing loss, cleft palate, and ocular complications, including myopia and retinal detachment (Dahiya et al., 2000). Radiographic features of SEDC typically include flattened and oval-shaped vertebral bodies, scoliosis, absence of ossification at the epiphyseal ends of the femoral heads, and irregular dysplasia of the femoral neck (Anderson et al., 1990). Other common findings include a flattened acetabular crest, deformities of the hip and knee joints, and disruptions in the ossification of long bones (Anderson et al., 1990).

SEDC is commonly associated with mutations in the *COL2A1* gene (Anderson et al., 1990). In their study, Zhang et al. reported that among 87 identified cases of SEDC, the majority of mutations were missense mutations (80 cases), with 57 of these occurring at the Gly site of the Gly-X-Y repeat sequence of the protein (Zhang et al., 2020). Additionally, the p.Arg989Cys site was identified as a potential mutation hotspot, indicating its significance in the pathogenesis of the disorder (Silveira et al., 2015).

##### 4.1.1.2 Czech dysplasia

Czech dysplasia is an autosomal dominant skeletal disorder characterized by early-onset progressive OA, normal stature, and brachydactyly, most commonly affecting the third and fourth toes (Burrage et al., 2013). Other features include mild platyspondyly with atypical vertebral endplates, progressive hearing loss, and the absence of cleft palate or ophthalmologic abnormalities (Burrage et al., 2013; Hoornaert et al., 2007). Radiographic findings often reveal short metacarpals and metatarsals, reduced intervertebral spaces, and the presence of osteochondromatosis (Burrage et al., 2013) or synovial osteochondromatosis (Moreira et al., 2023).

In cases where typical clinical features are absent but a family history of early-onset arthritis is present, WES can provide valuable diagnostic insights. Czech dysplasia is distinguishable from other type II collagenopathies by a single missense mutation in the *COL2A1* gene (R275C, c.823C > T) (Matsui et al., 2009). A previous report described a 3.5-year-old female patient with a family history of early-onset arthritis who presented only with prominent knees (Burrage et al., 2013). WES identified a c. 823C > T (p.R275C) mutation in the *COL2A1* gene, confirming the diagnosis of Czech dysplasia (Burrage et al., 2013). This case illustrates how WES enables early diagnosis in the presymptomatic stage, facilitating anticipatory guidance and genetic counseling (Burrage et al., 2013).

The two patients described in this study presented with bilateral hip pain, and imaging revealed acetabular dysplasia. However, no typical spinal abnormalities associated with SED, such as flattened vertebrae or odontoid hypoplasia, were observed. Additionally, there were no signs of extra spinal involvement characteristic of SED, such as short tubular bones, absent epiphyseal ossification, cleft palate, ocular complications, or sensorineural hearing loss. Furthermore, neither patient exhibited the hallmark features of Czech dysplasia, such as shortened third and fourth toes. Based on these findings, SED can be excluded as a diagnosis for the two patients.

#### 4.1.2 EO-OA

EO-OA, also referred to as premature OA, is typically secondary to inflammatory conditions or biomechanical abnormalities, such as osteochondrodysplasia, and often presents as part of a syndrome (Aury-Landas et al., 2016). Idiopathic EO-OA, which results from congenital or hereditary factors rather than trauma or infection, is rare, particularly in non-syndromic families without associated dysplasia or other underlying pathologies (Aury-Landas et al., 2016). The clinical features of EO-OA include the development of distal interphalangeal osteophytes, known as Heberden’s nodes, and progressive cartilage degeneration in the knees, hips, and other joints, and affected individuals frequently experience intermittent joint pain and swelling (Ala-Kokko et al., 1990). In previous studies, the average age of onset for EO-OA was reported to be 19 years (Rukavina et al., 2014).

Mutations in the *COL2A1* gene have been confirmed as a cause of EO-OA (Husar-Memmer et al., 2013). In 1995, one of 45 patients with familial EO-OA was identified as carrying a *COL2A1* mutation (Ritvaniemi et al., 1995). Additionally, four EO-OA probands were found to carry the same mutation (p. Arg275Cys) in the *COL2A1* gene (Carlson et al., 2006; Hoornaert et al., 2006; Lopponen et al., 2004; Williams et al., 1993).

According to established diagnostic criteria for EO-OA, which include radiographic evidence of OA, a body mass index (BMI) ≤30, an age of onset ≤50 years, and involvement of at least one joint site, the proband in this study meets the diagnostic criteria for EO-OA (Aury-Landas et al., 2016). However, the EO-OA observed in the proband is likely secondary to acetabular dysplasia and SC, rather than idiopathic EO-OA, given the underlying biomechanical abnormalities in the hip joints.

### 4.2 Discussion on differential diagnoses

Adult DDH must be differentiated from other conditions that cause hip pain, such as avascular necrosis of the femoral head (ANFH), hip OA, and ankylosing spondylitis (AS). When imaging reveals multiple loose bodies, it is essential to distinguish SC-associated findings from pigmented villonodular synovitis (PVNS).

When AD is accompanied by cystic degeneration of the femoral head, it should be differentiated from ANFH (Deng, 2014). Cystic degeneration in AD develops secondary to the underlying biomechanical abnormalities and often involves both the femoral head and the acetabulum, particularly in the weight-bearing areas (Deng, 2014). These cysts are typically adjacent to the joint surface, with smooth, well-defined margins and sclerotic edges. In some cases, a vacuum phenomenon may be observed (Deng, 2014). The morphology of the femoral head in AD remains largely preserved, though joint space narrowing and joint subluxation are common (Deng, 2014). In contrast, ANFH primarily affects the femoral head, with well-developed hip sockets and cystic degeneration restricted to the femoral head (Liu et al., 2010). Acetabular involvement is uncommon in ANFH, and the cystic lesions often exhibit mixed densities, poorly defined boundaries, and variable sizes, sometimes appearing as crescent-shaped or fissured areas (Liu et al., 2010). In advanced stages, the femoral head may fracture, collapse, and deform, with joint space narrowing occurring only in later stages (Deng, 2014). Joint subluxation is rare in ANFH, and patients often have a history of corticosteroid use, alcohol abuse, or trauma (Deng, 2014).

In late stages, AD may lead to secondary OA, which requires differentiation from primary degenerative hip OA. While both conditions share similar pathological mechanisms, the presence of AD serves as a precursor in secondary OA (Deng, 2014). AD-related OA tends to present at a younger age and is more common in females (Deng, 2014). In such cases, osteogenesis, sclerosis, and cystic degeneration predominantly affect the acetabulum, with larger cystic lesions and common joint subluxation (Deng, 2014). Conversely, primary degenerative OA is associated with normal acetabular development, occurs more frequently in males, and is characterized by bone hyperplasia and smaller cystic changes primarily involving the femoral head. In primary OA, the cysts are often chiseled and small, and joint subluxation is uncommon (Deng, 2014).

AS is more common in adolescents and mainly affects axial joints, particularly the sacroiliac joints and spine, often causing structural changes and strongly associated with the *HLA-B27* (Pedersen and Maksymowych, 2019). The proband sought medical attention due to bilateral hip pain, and an initial MRI of the sacroiliac joints revealed findings suggestive of sacroiliitis, which may have led the attending physician to consider AS as a possible diagnosis. However, it is essential to carefully evaluate the patient’s symptoms, laboratory findings, and imaging data to arrive at an accurate diagnosis. From a symptomatic perspective, low back pain is a hallmark feature of AS, often presenting early in the disease. However, in the proband’s case, low back pain was not a predominant symptom, and he did not report severe nocturnal pain. His symptoms worsened with activity and did not improve with rest, which is inconsistent with inflammatory back pain typically seen in AS. Additionally, the patient tested negative for *HLA-B27*, further reducing the likelihood of an AS diagnosis. The imaging findings also did not support AS. Although MRI initially indicated “sacroiliitis,” further analysis revealed that the sacroiliac joint surfaces were relatively smooth, and the observed inflammatory edema signal was inconsistent with sacroiliac arthritis. The sacroiliitis-like changes might instead be attributed to mechanical stress on the sacroiliac joints, potentially caused by prolonged use of crutches and poor posture, such as leaning forward. Therefore, a diagnosis of AS or spondyloarthritis could not be confirmed.

PVNS, also known as tenosynovial giant cell tumor (TGCT), is a benign lesion characterized by synovial villous nodular hyperplasia and hemosiderin deposition (Li et al., 2021). Malignant transformation and metastasis of PVNS are rare (Li et al., 2021). The clinical presentations of PVNS and SC are similar, with both conditions initially manifesting as hip pain and reduced range of motion (Startzman et al., 2016). Conservative treatment often fails to relieve symptoms in either disease (Xie et al., 2015). However, imaging studies can help differentiate the two conditions. PVNS is typically characterized by a slightly high-density soft tissue mass around the joint on CT, with varying degrees of adjacent bone destruction. The soft tissue mass in PVNS is often separated by fibrous septa (Han et al., 2010). In contrast, the CT scan of the proband’s hip joint revealed multiple high-density foci consistent with calcified nodules, without any evidence of bone destruction. These findings effectively ruled out PVNS as a diagnosis in this case.

### 4.3 Discussion on research progress regarding the genetics of DDH and its role in early diagnosis and treatment

Although extensive research has explored the genetic underpinnings of DDH, no definitive susceptibility genes have been identified (Wen et al., 2023). In a systematic review, Wen et al. pointed out that the genes most closely associated with DDH are *CX3CR1*, *ASPN*, *COL1A1*, *HOX*, and *GDF5*, with *GDF5* receiving the most attention in the literature (Wen et al., 2023). In addition to these well-studied susceptibility genes, Wen et al. also summarized that animal models have implicated other genes, including *TENM3*, *UFSP2*, and *WISP2* in the development of DDH (Wen et al., 2023). Futhermore, epigenetic changes also contribute to the pathogenesis of DDH. According to Wen et al., these include abnormal methylation of gene promoters, the altered expression of microRNAs such as miR-1-3p, miR-129-5p, and miR-140, and dysregulation of the long non-coding RNA (lncRNA) H19 (Wen et al., 2023).

Researchers have proposed that two distinct genetic pathways may underlie the development of DDH. The first involves genes responsible for the formation and development of acetabular cartilage and bone, while the second regulates genes involved in the development of the hip joint capsule and surrounding soft tissues (Wynne-Davies, 1970). Current research primarily focuses on these two pathways to better understand the molecular regulatory mechanisms involved in DDH (Zhao et al., 2023). Thus, identifying these genetic patterns and susceptibility genes is essential for clarifying the complex pathogenesis of DDH and holds potential for improving early detection, diagnosis, and treatment (Zhao et al., 2023). Advances in genetic research have highlighted the importance of identifying candidate and susceptibility genes for DDH (Zhao et al., 2023). In parallel, the development of genetic screening tools, including intrauterine diagnostic techniques, offers promising avenues for early detection and prenatal diagnosis (Zhao et al., 2023). Early genetic diagnosis could facilitate timely interventions, potentially improving patient outcomes (Zhao et al., 2023).

For children with a high risk of DDH but without obvious symptoms, the risk of developing DDH can be assessed by detecting these specific gene mutations (Agenbag et al., 2020). The detection of the *COL2A1* gene can be done through direct cycle sequencing and NGS methods (Agenbag et al., 2020). Although the *COL2A1* gene contains 54 exons, direct cycle sequencing is still a viable method for variant detection due to its relatively small overall size (Agenbag et al., 2020). However, NGS offers a better choice in terms of efficiency and coverage. NGS can sequence one or more target genes or even the entire exon group at the same time, thereby accurately identifying gene mutations that lead to hereditary diseases (Agenbag et al., 2020).

The earlier DDH is detected, the better the treatment outcome (Wang et al., 2023). Studies have shown that the effectiveness of treating DDH at the age of 8 is not superior to not treating it (Thomas, 2015). Therefore, early detection and diagnosis are key to treatment. Clinical screening methods for DDH in newborns and infants include physical examination, ultrasonography at 6 weeks, and X-ray screening at 4–6 months (Ji et al., 2022). These clinical screening methods have a low detection rate for DDH patients who later need hip joint replacement surgery, and there is a risk of over-treatment (Paton, 2017). Therefore, genetic screening should be supplemented for high-risk infants (Ji et al., 2022). For children without related gene mutations, regular testing and physical correction should be adopted as much as possible to avoid excessive treatment; for children with related gene mutations, long-term follow-up monitoring should be carried out simultaneously with treatment to determine the abnormal development of the hip joint (Ji et al., 2022).

The main purpose of early treatment of DDH is to restore the concentric relationship between the acetabulum and the femoral head, usually based on non-surgical treatment (Wang et al., 2023). When the age increases or the closed reduction correction is not possible, surgical treatment is needed to improve gait, alleviate pain, and prolong the lifespan of the hip joint (Wang et al., 2023). Most untreated adult DDH has a poor prognosis, while early diagnosis and treatment can improve disease outcomes, reduce the probability of needing surgical treatment later, and alleviate the burden of the disease on patients, families, and society (Wang et al., 2023).

In the present case, secondary SC was associated with congenital acetabular dysplasia, affecting both the hip and knee joints. This report provides a concise overview of research advances in the genetics of SC.

### 4.4 Discussion on research progress in the genetics of SC

Extensive research has been conducted to understand the pathogenesis of SC. Traditionally, SC has been considered to result from chondroid metaplasia of the joint synovium (Brucher et al., 2014). However, several studies have highlighted a possible association between SC and chromosomal abnormalities or gene mutations. Sciot et al. reported a case of SC involving clonal chromosomal changes and proposed that SC could be classified as a true neoplastic disorder (Sciot et al., 1998). Similarly, Mertens et al. analyzed the cytogenetics of four SC cases and identified the loss of chromosomal band 10q26 and rearrangements in 1p13 and 12q13, further supporting the theory of clonal proliferation in SC (Mertens et al., 1996). In a study by Totoki et al., an FN1-ACVR2A in-frame gene fusion was identified in one case of SC during a genomic investigation of benign and malignant cartilaginous neoplasms (Totoki et al., 2014). When analyzing seven additional cases of SC, the same gene fusion was detected in one other case. Subsequent studies by Amary et al. (2019) and Agaram et al. (2020) confirmed the presence of FN1 and/or ACVR2A gene rearrangements in SC using fluorescence *in situ* hybridization (FISH) and RNA sequencing. Additionally, Fukutani et al. (2022) identified a homozygous mutation (C/C genotype) in the Gli1 gene (rs2228226 G > C) in two cases of SC involving the temporomandibular joint. These findings suggest a genetic component in the development of SC.

This study has some limitations. Firstly, clinical whole exome high-throughput sequencing cannot fully cover certain genes or gene regions that are highly repetitive, low complexity, or pseudogene regions. The detection scope does not include genomic structural variations (such as large heterozygous deletions, duplications, and inversion rearrangements), large heterozygous insertion mutations, or mutations located in regulatory regions or deep intronic regions. Secondly, although these imaging findings provided sufficient evidence to establish a definitive diagnosis of acetabular dysplasia with overlapping SC, unfortunately, surgical specimens were not collected for pathological confirmation.

## 5 Conclusion

SC is a rare disease characterized by nonspecific symptoms that complicate diagnosis, often resulting in delayed detection and prolonged patient suffering. Consequently, when assessing young and middle-aged men presenting with hip pain, rheumatologists should consider conditions beyond AS and conduct a thorough inquiry into the patient’s family history to facilitate accurate differential diagnosis. To date, *COL2A1* gene mutations have not been reported in cases of acetabular dysplasia with SC. This study provides new evidence linking *COL2A1* gene mutations to acetabular dysplasia with SC, demonstrating that NGS can enhance diagnostic precision. These findings expand the clinical spectrum of *COL2A1* mutations, introducing new phenotypes associated with this gene.

## Data Availability

Publicly available datasets were analyzed in this study. This data can be found here: The data that support the findings of this study are openly available in ClinVar databases at https://www.ncbi.nlm.nih.gov/clinvar/RCV003228897.3/, Variation record RCV003228897.3; Submission Accession SCV002754582.

## References

[B1] Adelani M. A. Wupperman R. M. Holt G. E. (2008). Benign synovial disorders. J. Am. Acad. Orthop. Surg. 16 (5), 268–275. 10.5435/00124635-200805000-00005 18460687

[B2] Agaram N. P. Zhang L. Dickson B. C. Swanson D. Sung Y. S. Panicek D. M. (2020). A molecular study of synovial chondromatosis. Genes Chromosom. Cancer 59 (3), 144–151. 10.1002/gcc.22812 31589790 PMC7147082

[B3] Agenbag G. Vorster A. Julius S. Ramesar R. Beighton P. (2020). Namaqualand hip dysplasia in South Africa: the molecular determinant elucidated. S Afr. Med. J. 111 (1), 57–60. 10.7196/SAMJ.2020.v111i1.14561 33404007

[B4] Ala-Kokko L. Baldwin C. T. Moskowitz R. W. Prockop D. J. (1990). Single base mutation in the type II procollagen gene (COL2A1) as a cause of primary osteoarthritis associated with a mild chondrodysplasia. Proc. Natl. Acad. Sci. U. S. A. 87 (17), 6565–6568. 10.1073/pnas.87.17.6565 1975693 PMC54577

[B5] Alexander J. E. Holder J. C. Mcconnell J. R. Fontenot E. Jr (1987). Synovial osteochondromatosis. Am. Fam. Physician 35 (2), 157–161. 3812170

[B6] Al Kaissi A. Ryabykh S. Pavlova O. M. Ochirova P. Kenis V. Chehida F. B. (2019). The Managment of cervical spine abnormalities in children with spondyloepiphyseal dysplasia congenita: observational study. Med. (Baltimore) 98 (1), e13780. 10.1097/MD.0000000000013780 PMC6344193 30608389

[B7] Amary F. Perez-Casanova L. Ye H. Cottone L. Strobl A. C. Cool P. (2019). Synovial chondromatosis and soft tissue chondroma: extraosseous cartilaginous tumor defined by FN1 gene rearrangement. Mod. Pathol. 32 (12), 1762–1771. 10.1038/s41379-019-0315-8 31273315 PMC6882679

[B8] Anderson I. J. Goldberg R. B. Marion R. W. Upholt W. B. Tsipouras P. (1990). Spondyloepiphyseal dysplasia congenita: genetic linkage to type II collagen (COL2AI). Am. J. Hum. Genet. 46 (5), 896–901. 1971141 PMC1683599

[B9] Aury-Landas J. Marcelli C. Leclercq S. Boumediene K. Bauge C. (2016). Genetic determinism of primary early-onset osteoarthritis. Trends Mol. Med. 22 (1), 38–52. 10.1016/j.molmed.2015.11.006 26691295

[B10] Barat-Houari M. Sarrabay G. Gatinois V. Fabre A. Dumont B. Genevieve D. (2016). Mutation update for COL2A1 gene variants associated with type II collagenopathies. Hum. Mutat. 37 (1), 7–15. 10.1002/humu.22915 26443184

[B11] Basit S. Albalawi A. M. Alharby E. Khoshhal K. I. (2017). Exome sequencing identified rare variants in genes HSPG2 and ATP2B4 in a family segregating developmental dysplasia of the hip. BMC Med. Genet. 18 (1), 34. 10.1186/s12881-017-0393-8 28327142 PMC5361705

[B12] Basit S. Hannan M. A. Khoshhal K. I. (2016). Developmental dysplasia of the hip: usefulness of next generation genomic tools for characterizing the underlying genes - a mini review. Clin. Genet. 90 (1), 16–20. 10.1111/cge.12755 26842108

[B13] Brucher N. Faruch-Bilfeld M. Molinier F. Brouchet-Gomez A. Lapegue F. Sans N. (2014). Primary synovial osteochondromatosis of the first interphalangeal joint of the foot: a case report. Diagn Interv. Imaging 95 (4), 451–453. 10.1016/j.diii.2013.10.003 24231342

[B14] Burrage L. C. Lu J. T. Liu D. S. Moss T. J. Gibbs R. Schlesinger A. E. (2013). Early childhood presentation of Czech dysplasia. Clin. Dysmorphol. 22 (2), 76–80. 10.1097/MCD.0b013e32835fff39 23448908 PMC3673284

[B15] Carlson K. M. Yamaga K. M. Reinker K. A. Hsia Y. E. Carpenter C. Abe L. M. (2006). Precocious osteoarthritis in a family with recurrent COL2A1 mutation. J. Rheumatol. 33 (6), 1133–1136. 16755660

[B16] Dahiya R. Cleveland S. Megerian C. A. (2000). Spondyloepiphyseal dysplasia congenita associated with conductive hearing loss. Ear Nose Throat J. 79 (3), 178–182. 10.1177/014556130007900312 10743764

[B17] Deng J. L. (2014). X-ray diagnosis and differential diagnosis of adult acetabular dysplasia. Orthop. J. China 22 (11), 971–975. 10.3977/j.issn.1005-8478.2014.11.03

[B18] Feng W. J. Wang H. Shen C. Zhu J. F. Chen X. D. (2017). Severe cartilage degeneration in patients with developmental dysplasia of the hip. IUBMB Life 69 (3), 179–187. 10.1002/iub.1606 28185391

[B19] Fukutani T. Toratani S. Kanda T. Matsui K. Yamasaki S. Sumi K. (2022). Two cases of temporomandibular synovial chondromatosis associated with Gli1 gene mutation. Int. J. Environ. Res. Public Health 19 (8), 4702. 10.3390/ijerph19084702 35457572 PMC9030668

[B20] Granchi D. Stea S. Sudanese A. Toni A. Baldini N. Giunti A. (2002). Association of two gene polymorphisms with osteoarthritis secondary to hip dysplasia. Clin. Orthop. Relat. Res. 403, 108–117. 10.1097/00003086-200210000-00018 12360016

[B21] Han H. Wang Y. Yin M. Xu H. (2010). Imaging manifestations of pigmented villonodular synovitis. Huazhong Univ. Sci. Technolog. Med. Sci. 39 (2), 258–260+271. 10.3870/j.issn.1672-0741.2010.02.028

[B22] Hildebrand B. (2015). Mutation in the type II collagen gene (COL2A1) as a cause of early osteoarthritis and juvenile idiopathic arthritis-A pseudorheumatoid arthritis variant. Semin. Arthritis Rheum. 44 (6), e22. 10.1016/j.semarthrit.2015.01.001 25686763

[B23] Hoornaert K. P. Dewinter C. Vereecke I. Beemer F. A. Courtens W. Fryer A. (2006). The phenotypic spectrum in patients with arginine to cysteine mutations in the COL2A1 gene. J. Med. Genet. 43 (5), 406–413. 10.1136/jmg.2005.035717 16155195 PMC2564515

[B24] Hoornaert K. P. Marik I. Kozlowski K. Cole T. Le Merrer M. Leroy J. G. (2007). Czech dysplasia metatarsal type: another type II collagen disorder. Eur. J. Hum. Genet. 15 (12), 1269–1275. 10.1038/sj.ejhg.5201913 17726487

[B25] Husar-Memmer E. Ekici A. Al Kaissi A. Sticht H. Manger B. Schett G. (2013). Premature osteoarthritis as presenting sign of type II collagenopathy: a case report and literature review. Semin. Arthritis Rheum. 42 (4), 355–360. 10.1016/j.semarthrit.2012.05.002 22717203

[B26] Ji C. Li C. W. Deng L. F. (2022). Research progress in the prevention and treatment of developmental dysplasia of the hip. Chin. J. Orthop. 42 (01), 54–64. 10.3760/cma.j.cn121113-20210914-00555

[B27] Kenanidis E. Gkekas N. K. Karasmani A. Anagnostis P. Christofilopoulos P. Tsiridis E. (2020). Genetic predisposition to developmental dysplasia of the hip. J. Arthroplasty 35 (1), 291–300.e1. 10.1016/j.arth.2019.08.031 31522852

[B28] Li H. Ma L. Wang B. Cui Y. Xiao T. (2015). Identification of a novel mutation of the COL2A1 gene in a Chinese family with spondyloepiphyseal dysplasia congenita. Eur. Spine J. 24 (8), 1813–1819. 10.1007/s00586-015-3999-6 25967556

[B29] Li W. Hu P. Bi S. (2021). Research progress of pigmented villonodular synovitis. Int. J. Orthop. Sci. 42 (01), 40–44. 10.3969/j.issn.1673-7083.2021.01.010

[B30] Liu J. P. Zhang S. Q. Chen W. H. (2010). Radiographic imaging feature and differential diagnosis of early femoral head necrosis. China J. Orthop. Traumatology 23 (5), 344–348. 10.3969/j.issn.1003-0034.2010.05.008 20575286

[B31] Liu L. (2014). “The clinical diagnosis and gene mutation analysis of spinal epiphyseal dysplasia,”. Master’s Thesis (Beijing: Peking Union Medical College).

[B32] Lopponen T. Korkko J. Lundan T. Seppanen U. Ignatius J. Kaariainen H. (2004). Childhood-onset osteoarthritis, tall stature, and sensorineural hearing loss associated with Arg75-Cys mutation in procollagen type II gene (COL2A1). Arthritis Rheum. 51 (6), 925–932. 10.1002/art.20817 15593085

[B33] Matsui Y. Michigami T. Tachikawa K. Yamazaki M. Kawabata H. Nishimura G. (2009). Czech dysplasia occurring in a Japanese family. Am. J. Med. Genet. A 149A (10), 2285–2289. 10.1002/ajmg.a.33010 19764028

[B34] Mertens F. Jonsson K. Willen H. Rydholm A. Kreicbergs A. Eriksson L. (1996). Chromosome rearrangements in synovial chondromatous lesions. Br. J. Cancer 74 (2), 251–254. 10.1038/bjc.1996.346 8688330 PMC2074582

[B35] Moreira L. A. Carvalho D. R. Santos S. C. L. Silva C. C. E. Ferreira B. S. A. Cunha B. M. D. (2023). Czech dysplasia mimicking rheumatoid arthritis: case series and literature review. Mod. Rheumatol. 34 (4), 705–710. 10.1093/mr/road070 37489771

[B36] Nandhagopal T. Tiwari V. De Cicco F. L. (2024). Developmental dysplasia of the hip. Treasure Island (FL): StatPearls Publishing. 33085304

[B37] Ortiz-Neira C. L. Paolucci E. O. Donnon T. (2012). A meta-analysis of common risk factors associated with the diagnosis of developmental dysplasia of the hip in newborns. Eur. J. Radiol. 81 (3), e344–e351. 10.1016/j.ejrad.2011.11.003 22119556

[B38] Paton R. W. (2017). Screening in developmental dysplasia of the hip (DDH). Surgeon 15 (5), 290–296. 10.1016/j.surge.2017.05.002 28619546

[B39] Pedersen S. J. Maksymowych W. P. (2019). The pathogenesis of ankylosing spondylitis: an update. Curr. Rheumatol. Rep. 21 (10), 58. 10.1007/s11926-019-0856-3 31712904

[B40] Ritvaniemi P. Korkko J. Bonaventure J. Vikkula M. Hyland J. Paassilta P. (1995). Identification of COL2A1 gene mutations in patients with chondrodysplasias and familial osteoarthritis. Arthritis Rheum. 38 (7), 999–1004. 10.1002/art.1780380717 7612049

[B41] Rukavina I. Mortier G. Van Laer L. Frkovic M. Dapic T. Jelusic M. (2014). Mutation in the type II collagen gene (COL2AI) as a cause of primary osteoarthritis associated with mild spondyloepiphyseal involvement. Semin. Arthritis Rheum. 44 (1), 101–104. 10.1016/j.semarthrit.2014.03.003 24681029

[B42] Sciot R. Dal Cin P. Bellemans J. Samson I. Van Den Berghe H. Van Damme B. (1998). Synovial chondromatosis: clonal chromosome changes provide further evidence for a neoplastic disorder. Virchows Arch. 433 (2), 189–191. 10.1007/s004280050235 9737798

[B43] Sher C. Ramesar R. Martell R. Learmonth I. Tsipouras P. Beighton P. (1991). Mild spondyloepiphyseal dysplasia (Namaqualand type): genetic linkage to the type II collagen gene COL2A1. Am. J. Hum. Genet. 48 (3), 518–524. 1671807 PMC1682978

[B44] Silveira K. C. Bonadia L. C. Superti-Furga A. Bertola D. R. Jorge A. A. Cavalcanti D. P. (2015). Six additional cases of SEDC due to the same and recurrent R989C mutation in the COL2A1 gene–the clinical and radiological follow-up. Am. J. Med. Genet. A 167 (4), 894–901. 10.1002/ajmg.a.36954 25735649

[B45] Startzman A. Collins D. Carreira D. (2016). A systematic literature review of synovial chondromatosis and pigmented villonodular synovitis of the hip. Phys. Sportsmed. 44 (4), 425–431. 10.1080/00913847.2016.1216238 27462929

[B46] Thomas S. R. (2015). A review of long-term outcomes for late presenting developmental hip dysplasia. Bone Jt. J. 97 (6), 729–733. 10.1302/0301-620X.97B6.35395 26033050

[B47] Tian J. Bi W. Meng F. (2003). Imaging diagnosis of acetabular dysplastic coxarthrosis in adult. Chin. J. Radiol. 37 (02), 135–139.

[B48] Tofield C. E. Mackinnon C. A. (2003). Cleft palate repair in spondyloepiphyseal dysplasia congenita: minimizing the risk of cervical cord compression. Cleft Palate Craniofac J. 40 (6), 629–631. 10.1597/02-159 14577812

[B49] Totoki Y. Yoshida A. Hosoda F. Nakamura H. Hama N. Ogura K. (2014). Unique mutation portraits and frequent COL2A1 gene alteration in chondrosarcoma. Genome Res. 24 (9), 1411–1420. 10.1101/gr.160598.113 25024164 PMC4158757

[B50] Wang K. Z. Zhou J. S. Shi Z. J. Hu Y. C. Zhang H. Chen X. D. (2023). Guideline for the diagnosis and treatment of developmental dysplasia of the hip in China (2023 edition). Chin. J. Anat. Clin. 28 (8), 493–511. 10.3760/cma.j.cn101202-20230612-00145

[B51] Warman M. L. Cormier-Daire V. Hall C. Krakow D. Lachman R. Lemerrer M. (2011). Nosology and classification of genetic skeletal disorders: 2010 revision. Am. J. Med. Genet. A 155A (5), 943–968. 10.1002/ajmg.a.33909 21438135 PMC3166781

[B52] Wen J. Ping H. Kong X. Chai W. (2023). Developmental dysplasia of the hip: a systematic review of susceptibility genes and epigenetics. Gene 853, 147067. 10.1016/j.gene.2022.147067 36435507

[B53] Williams C. J. Considine E. L. Knowlton R. G. Reginato A. Neumann G. Harrison D. (1993). Spondyloepiphyseal dysplasia and precocious osteoarthritis in a family with an Arg75-->Cys mutation in the procollagen type II gene (COL2A1). Hum. Genet. 92 (5), 499–505. 10.1007/BF00216458 8244341

[B54] Wynne-Davies R. (1970). Acetabular dysplasia and familial joint laxity: two etiological factors in congenital dislocation of the hip. A review of 589 patients and their families. J. Bone Jt. Surg. Br. 52 (4), 704–716. 10.1302/0301-620x.52b4.704 5487570

[B55] Xie G. P. Jiang N. Liang C. X. Zeng J. C. Chen Z. Y. Xu Q. (2015). Pigmented villonodular synovitis: a retrospective multicenter study of 237 cases. PLoS One 10 (3), e0121451. 10.1371/journal.pone.0121451 25799575 PMC4370558

[B56] Zamborsky R. Kokavec M. Harsanyi S. Attia D. Danisovic L. (2019). Developmental dysplasia of hip: perspectives in genetic screening. Med. Sci. Basel 7 (4), 59. 10.3390/medsci7040059 30979092 PMC6524033

[B57] Zhang B. Zhang Y. Wu N. Li J. Liu H. Wang J. (2020). Integrated analysis of COL2A1 variant data and classification of type II collagenopathies. Clin. Genet. 97 (3), 383–395. 10.1111/cge.13680 31758797

[B58] Zhao Z. Xiong Z. Zeng S. (2023). Research progress on the genetics and molecular regulatory mechanisms of developmental hip dysplasia. Clin. Ped Sur 22 (11), 1095–1100. 10.3760/cma.j.cn101785-202210022-019

